# Invasion Genetics of the Horse-Chestnut Leaf Miner, *Cameraria ohridella* (Lepidoptera: Gracillariidae), in European Russia: A Case of Successful Involvement of Citizen Science in Studying an Alien Insect Pest

**DOI:** 10.3390/insects14020117

**Published:** 2023-01-24

**Authors:** Natalia I. Kirichenko, Natalia N. Karpun, Elena N. Zhuravleva, Elena I. Shoshina, Vasily V. Anikin, Dmitrii L. Musolin

**Affiliations:** 1 Sukachev Institute of Forest, Siberian Branch of the Russian Academy of Sciences, Federal Research Center ‘Krasnoyarsk Science Center SB RAS’, Akademgorodok 50/28, 660036 Krasnoyarsk, Russia; 2 Institute of Ecology and Geography, Siberian Federal University, Svobodny pr. 79, 660041 Krasnoyarsk, Russia; 3 Federal Research Centre the Subtropical Scientific Centre of the Russian Academy of Sciences, Yana Fabritsiusa Street 2/28, 354002 Sochi, Russia; 4 Department of Forest Protection, Wood Science and Game Management, Saint Petersburg State Forest Technical University, Institutskiy per. 5, 194021 Saint Petersburg, Russia; 5 Department of Animal Morphology and Ecology, Chernyshevsky Saratov State University, Astrakhanskaya Street 83, 410012 Saratov, Russia; 6 European and Mediterranean Plant Protection Organization (EPPO/OEPP), 21 boulevard Richard Lenoir, 75011 Paris, France

**Keywords:** leaf-mining moth, invasion, European Russia, citizen science, DNA barcoding, phylogeography, *Aesculus*, *Acer*, damage level

## Abstract

**Simple Summary:**

The horse-chestnut leaf miner, *Cameraria ohridella* Deschka and Dimić, 1986 (Lepidoptera: Gracillariidae), is an ornamental invasive alien insect pest that invaded Russia in the beginning of the 21st century. With the help of volunteers, we clarified the secondary range of species in the European part of Russia and performed extensive sampling of insect specimens for molecular genetic analysis. The pest was confirmed in 24 administrative regions in the European part of Russia. In southern Russia, it produced spectacular outbreaks in 2021. The DNA barcoding data obtained for 201 specimens from European Russia indicates the presence of two haplotypes, which are also known from the secondary range of this species in Eastern and Western Europe.

**Abstract:**

Based on the intensive monitoring conducted by our team and volunteers in 2021, the secondary range of an alien horse-chestnut leaf miner, *Cameraria ohridella* Deschka & Dimić, 1986 (Lepidoptera: Gracillariidae), was specified in European Russia. This invasive pest was confirmed in 24 out of 58 administrative regions of Russia, which it has occupied for approximately 16 years. Analysis of the COI mtDNA gene sequenced in 201 specimens collected in 21 regions of the European part of Russia indicates the occurrence of two haplotypes (A and B), which are also present in the secondary range of *C. ohridella* in Eastern and Western Europe. The haplotype A dominated and was present in 87.5% of specimens from European Russia. In 2021, *C. ohridella* produced spectacular outbreaks in *Aesculus hippocastanum* in southern Russia, where it damaged more than 50% of the leaves in trees in 24 out of 30 distant localities. In the south of the country, the pest infested *Acer pseudoplatanus*, whereas other species of *Acer* of European, East Asian, and North American origin showed no signs of attacks. Taking into account that *Ae. hippocastanum* is present in most regions of European Russia, we expect a further range expansion of *C. ohridella* up to the Ural Mountains.

## 1. Introduction

Invasions of herbivorous insects are one of the critical issues of modern ecology that is attracting more and more attention of specialists and practitioners [1,2,3]. Alien species expand their ranges and penetrate into new regions, causing cascades of ecological and economic problems [4,5,6,7]. Notably, in their native ranges, such species often have hardly any significant impact on local ecosystems, whereas in the invaded regions, they can become serious pests [8]. Invasions of herbivorous insects are increasingly facilitated by the exchange of goods (including live plants and plant materials) between regions, countries, and continents [9,10,11]. Climate change, as well as a pronounced transformation of ecosystems (often towards their simplification), also create a favorable environment for invasive alien species [12,13,14]. Altogether, these factors favor the unintentional introduction, establishment, and range expansion of alien insect species in new areas.

Urban forests, botanical gardens, parks, alleys, and roadside plantings play an invaluable role in the functioning of man-made ecosystems [15,16]. At the same time, such plantings can be significantly affected by alien pests; furthermore, they can serve as reservoirs and corridors for further spreading of alien species [15,17]. As a result of the introduction of ornamental plants (in particular, the import of plant seedlings) and an increasing traffic flow (with vehicles as vectors), alien insect species continue to penetrate and spread in the European part of Russia [18,19]. This part of the country, especially its southern territories (including the Black Sea coast), is a recipient region of many alien herbivorous insect pests, which penetrate and settle here and then continue spreading into neighboring regions and countries [19,20]. 

Leaf-mining insects, whose larvae live inside leaf blades, can notably damage trees and shrubs in urban plantings [21,22]. Significant damage by leaf miners leads to premature drying and falling of leaves, which negatively affects both plant health and aesthetics of the local landscapes [22,23,24]. 

Among leaf-mining insects, the horse-chestnut leaf miner, *Cameraria ohridella* Deschka & Dimić, 1986 (Lepidoptera: Gracillariidae), is one of the most curious examples of invasion in Europe and the European part of Russia [25]. In the last few decades, this species has spread across most of Europe from its original, relatively small native range (the Balkans), which was clarified by a molecular genetic analysis [26,27]. The insect severely damages horse chestnut, *Aesculus hippocastanum* L. (Sapindaceae), a tree that is actively used in landscaping in Europe [28], including European Russia [29,30]. The moth is able to outbreak within a short period, and its foci can function for years [22]. The pest was recorded for the first time in the European part of Russia in 2003 (in Kaliningrad, an enclaved region on the Baltic Sea coast); two years later, it was documented in Moscow [31,32]. Since then, it has continued spreading across European Russia, causing notable damage to its host plant [30,33,34]. 

Citizen science, or volunteer monitoring conducted with the participation of citizens, is being increasingly used in detecting invasive alien species (including insects). Clarifying their secondary ranges and sampling requires data for further analysis by researchers [35,36,37,38,39]. This tool is especially important when it comes to the inspection of vast territories with regard to the presence of alien species. With the involvement of citizen science, here, we explored the invasion range of *C. ohridella* and studied the genetic variability of its populations in the European part of Russia. Additionally, we estimated the damage caused by this alien pest to *Ae. hippocastanum* plantings and clarified the trophic relationships with other plants of the family Sapindaceae, such as *Acer* spp., bearing in mind that exotic *Acer* species (including those of North American and East Asian origin) are widely used in landscaping in Russia.

## 2. Materials and Methods

### 2.1. Study Area

The study was carried out in the European part of Russia in 39 administrative regions. To designate administrative units, the following terms are used below: “Oblast” for a Province and “Krai” for an Administrative Territory. The field surveys were carried in Arkhangelsk, Astrakhan, Belgorod, Bryansk, Chelyabinsk, Izhevsk, Kaliningrad, Kirov, Kostroma, Kurgan, Leningrad, Moscow, Nizhny Novgorod, Novgorod, Orenburg, Perm, Rostov, Sverdlovsk, Tula, Vologda, Yaroslavl Oblasts, Krasnodar and Stavropol Krais, Adygea, Bashkortostan, Chuvashia, Crimea, Dagestan, Kabardino-Balkaria, Kalmykia, Karachay-Cherkessia, Komi, Mari El, Mordovia, and Tatarstan Republics in June–August 2021. As an exception, sampling in Penza, Samara, Saratov, and Ulyanovsk Oblasts was performed in July–October 2020. In Russia, *C. ohridella* specimens were searched and collected mainly in the major cities (as the horse chestnuts are mostly present in urbanized areas) and, to a lesser extent, in smaller settlements.

In southern Russia, the study was performed by our team in 30 localities of 5 administrative regions: Krasnodar (19 localities) and Stavropol (8) Krais, the Republics of Adygea, Kabardino-Balkaria, and Karachay-Cherkessia (1 in each); and in 11 localities of 5 administrative regions of the Volga Region: Samara (1), Saratov (5), Penza (1), and Ulyanovsk (4) Oblasts. The other 34 administrative regions were examined by volunteers.

### 2.2. Volunteers’ Involvement

The sampling protocol prepared for volunteers was distributed through social networks and messaging apps (Facebook, Instagram, WhatsApp, Telegram, Viber, VKontakte) and through mail circulation targeting specialists (through conference and symposium mailing lists) and non-specialist communities across the European part of Russia. The volunteers were asked to check the leaves of horse chestnuts and maples in their villages, towns, and cities for the presence of typical mines of *C. ohridella* in the period from the middle of June to the middle of August 2021 (which corresponds to the presence of the first and second generations). They were also advised to make high-resolution photographs of damaged leaves and record GPS coordinates of findings and email the data to us to confirm the species in the sampled localities. Furthermore, the volunteers were asked to randomly sample 10–20 leaves with characteristic mines from up to 5 different trees of *Ae. hippocastanum* and *Acer* spp. in the lower part of the tree crown, herbarize them for 2–3 days (to decrease the probability of molding), pack them in absorbent paper tissue with a label (sampled locality, coordinates, host plant, date, and collector’s name), and dispatch them to us by post. In all cases, posting expenditures were covered by our team for simplifying the specimen delivery to our laboratory for further molecular genetic study. 

### 2.3. Insect Specimen Sampling

In southern Russia and in the Volga Region, we randomly sampled 10–20 leaves (depending on their size and their damage) with the mines of *C. ohridella* from 3–5 horse-chestnut trees and maples in the lower part of the tree crown. The leaves were packed in zip bags with a field label and delivered to the laboratory.

Larvae and pupae were dissected from the leaves; the mines were opened with a sterile needle to find larvae or pupae of *C. ohridella*, which served as material for molecular genetic study. All insect specimens collected from the leaf mines were photographed with Xiaomi 11 Lite (Xiaomi Inc., Beijing, China). A few pupae of *C. ohridella* were reared to the adult stage for the species’ confirmation based on the adult morphology [40]. The collected leaves with the mines of *C. ohridella* were placed in an annotated herbarium collection and stored in the laboratory of Forest Zoology of V.N. Sukachev Institute of Forest SB RAS (Krasnoyarsk, Russia).

### 2.4. Molecular Genetic Analysis

Larvae, pupae, and adult moths (3–5 specimens from each locality) were placed into genetic microplates (BOLD System, CCDB, ON, Canada) with a drop of 95% ethanol. To avoid cross-contamination when transferring insects to the wells, forceps were washed in 95% ethanol, and their tips were treated with the flame of a spirit lamp after each manipulation. Before DNA barcoding, the plates were stored in a freezer at –20°C to prevent DNA degradation.

Overall, 201 specimens of *C. ohridella* (156 larvae, 41 pupae, and 4 adults) sampled in 21 regions of European Russia were DNA barcoded (Table S1). In the specimens, the mitochondrial cytochrome oxidase I gene (COI mtDNA, 658 bp) was sequenced using the set of primers C_LepFolF/C_LepFolR, following the standard high-throughput protocol [41]. DNA barcoding was performed in the Canadian Center for DNA barcoding (CCDB) at the University of Guelph (Canada). The specimen data are provided in Table S1. The sequences, biogeographic data, and images of the vouchers were deposited in the Barcode of Life Data Systems (BOLD) [42] and the National Centre for Biotechnology Information (NCBI) and accessed using the link: dx.doi.org/10.5883/DS-CAMRUS (accessed on: 24 January 2023).

For comparative analysis, six published and freely available DNA barcodes of *C. ohridella* were borrowed from BOLD: Macedonia (1 sequence) and Greece (1), Hungary (1) and France (1), and Belarus (2) [25,26]. Macedonia and Greece, together with other Balkan countries, are considered to be a primary range of *C. ohridella* [25,26].

The DNA barcodes were aligned using the BioEdit 7.1.7 program [43]. The phylogenetic tree was built with the maximum likelihood approach using the Kimura Two-ParameterModel and the bootstrap analysis with 2000 iterations in the MEGA X program [44]. The sequences were assigned to a certain haplotype by comparing them with a set of haplotypes identified in the primary and secondary range of *C. ohridella* in Eastern and Western Europe [25]. Two sequences of the related East Asian species, *Cameraria niphonica* Kumata, 1963, whose larvae we sampled from *Acer pseudosieboldianum* and *A. caudatum* Wall. in the Russian Far East (Primorsky Krai), were used for rooting the genetic tree. 

Sampling region and possible range expansion were illustrated using Google Maps, and the geographic distribution of COI haplotypes was displayed with an altitudinal background using ArcGIS 9.3 [45].

### 2.5. Trophic Associations

To clarify the trophic relationships of *C. ohridella*, we examined trees of *Ae. hippocastanum* and *Acer* spp. in the landscaping of the regions of southern Russia: Krasnodar and Stavropol Krais, the Republics of Adygea, Kabardino-Balkaria, and Karachay-Cherkessia. Examination was performed by the same researchers among the coauthors to maintain the same search efforts and method. The data on leaf damage occasionally estimated by the volunteers in a few regions were not involved in this analysis. The following *Acer* species were surveyed: *A. platanoides* L. (natural range: Europe, the European part of Russia (middle zone), *A. pseudoplatanus* L. (Central Europe, Southwest Asia, Caucasus), *A. pictum* Thunb., *A. ginnala* Maxim., *A. palmatum* Thunb. (East Asia), and *A. negundo* L. (North America). 

On trees, the leaves were examined on all lower branches at a height of 1–2 m from the soil surface in all directions for the presence of upper-side spot-like mines characteristic of *C. ohridella.* Between 3 to 10 trees of each species were involved in the survey. The findings of the mines were documented; all mines found on *Acer* spp. were collected in the herbarium.

In the same spots, 3–15 horse-chestnut trees were further examined: leaves on four branches were checked for the presence of mines of *C. ohridella* using the same approach as the inspection for *Acer* spp. The plant damage level was documented at each locality from 9 to 11 August 2021, which corresponded to the end of the first generation and the beginning of the second generation of *C. ohridella*. The following levels of damage were taken into account: low (a relative number of leaves with mines in the lower part of the crown: 1–25%), moderate (26–50%), high (51–75%), and severe (>75%).

## 3. Results

### 3.1. Sampling Efforts and the Detection of C. ohridella across the European Part of Russia

**Figure 1:**
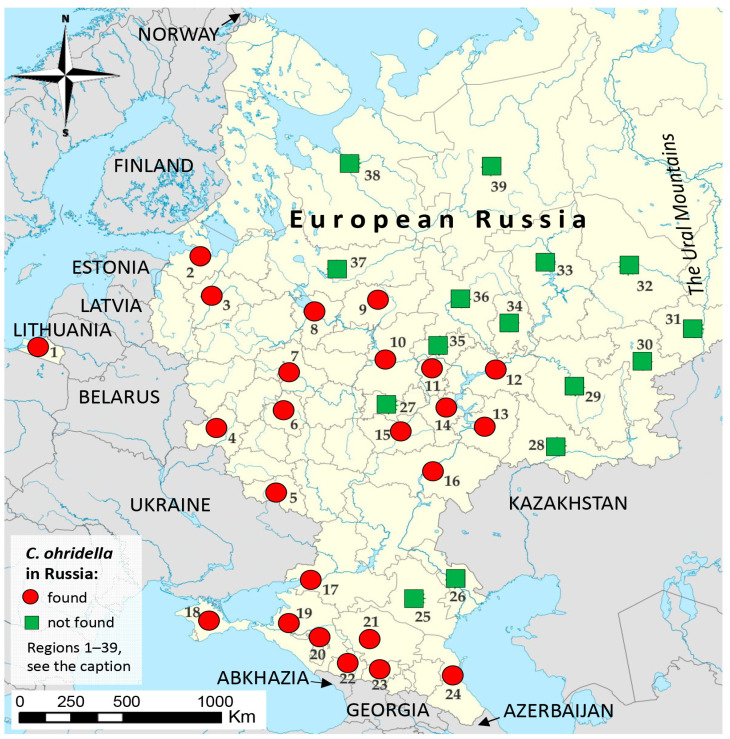
The detection of *Cameraria ohridella* in the European part of Russia based on the monitoring conducted by our team and volunteers in 2021. Administrative regions indicated on the map: 1—Kaliningrad, 2—Leningrad, 3—Novgorod, 4—Bryansk, 5—Belgorod, 6—Tula, 7—Moscow, 8—Yaroslavl, 9—Kostroma, 10—Nizhny Novgorod Oblasts, 11—Chuvashia, 12—Tatarstan Republics, 13—Samara, 14—Ulyanovsk, 15—Penza, 16—Saratov, 17—Rostov Oblasts, 18—Crimea Republic, 19—Krasnodar Krai, 20—Adygea Republic, 21—Stavropol Krai, 22—Karachay-Cherkessia, 23—Kabardino-Balkaria, 24—Dagestan, 25—Kalmykia Republics, 26—Astrakhan Oblast, 27—Mordovia Republic, 28—Orenburg Oblast, 29—Bashkortostan Republic, 30—Chelyabinsk, 31—Kurgan, 32—Sverdlovsk, 33—Perm, 34—Izhevsk Oblasts, 35—Mari El Republic, 36—Kirov, 37—Vologda, 38—Arkhangelsk Oblasts, 39—Komi Republic. The map is built based on an image from © Google, 2022.

Overall, in the European part of Russia, 39 out of 58 administrative regions (i.e., 67% of all regions in this part of Russia) were surveyed for the presence of *C. ohridella* (Figure 1). Our team sampled nine regions: Stavropol and Krasnodar Krais, the Republics of Adygea, Kabardino-Balkaria, Karachay-Cherkessia, Penza, Samara, Saratov, and Ulyanovsk Oblasts. The other 30 administrative regions were examined by volunteers.

Overall, 89 replies were received from citizens from the European part of Russia through social networks, messaging apps, and email systems. The images of leaves with characteristic damage and the specimens of *C. ohridella* were obtained in 58 replies of volunteers from 15 administrative regions of Russia, confirming the presence of *C. ohridella* in Belgorod, Bryansk, Kaliningrad, Kostroma, Leningrad, Moscow, Novgorod, Nizhny Novgorod, Rostov, Tula, Yaroslavl Oblasts, Crimea, Dagestan, Chuvashia, and Tatarstan Republics (Figure 1).

Furthermore, 31 replies arrived from other 15 regions: Arkhangelsk, Astrakhan, Chelyabinsk, Izhevsk, Kirov, Kurgan, Orenburg, Perm, Sverdlovsk, Vologda Oblasts, Bashkortostan, Kalmykia, Komi, Mordovia, and Mari El Republics, reporting the absence of *C. ohridella* in urban plantings in spite of intensive search.

Thus, overall, *C. ohridella* was confirmed in 24 administrative regions of European Russia, i.e., in 15 regions from which positive replies arrived from volunteers, plus 9 regions examined by our team.

### 3.2. Genetic Variability of C. ohridella in the European Part of Russia

A total of 201 DNA barcodes were obtained from *C. ohridella* specimens sampled in 21 regions of the European part of Russia, i.e., in the northwest (Kaliningrad, Leningrad Oblasts), center (Belgorod, Bryansk, Moscow, Novgorod, Penza, Rostov, Samara, Saratov, Tula, Ulyanovsk, Yaroslavl Oblasts), and south (Stavropol and Krasnodar Krais, Adygea, Crimea, Chuvashia, Dagestan, Kabardino-Balkaria, Karachay-Cherkessia Republics). Overall, 175 COI sequences (93%) yielded the targeted length of 658 bp. The other 15 sequences (i.e., 7%) had a shorter length, from 612 to 652 bp. All sequences were assigned in BOLD to a unique BIN of *C. ohridella*, BOLD:AAA1236.

**Figure 2:**
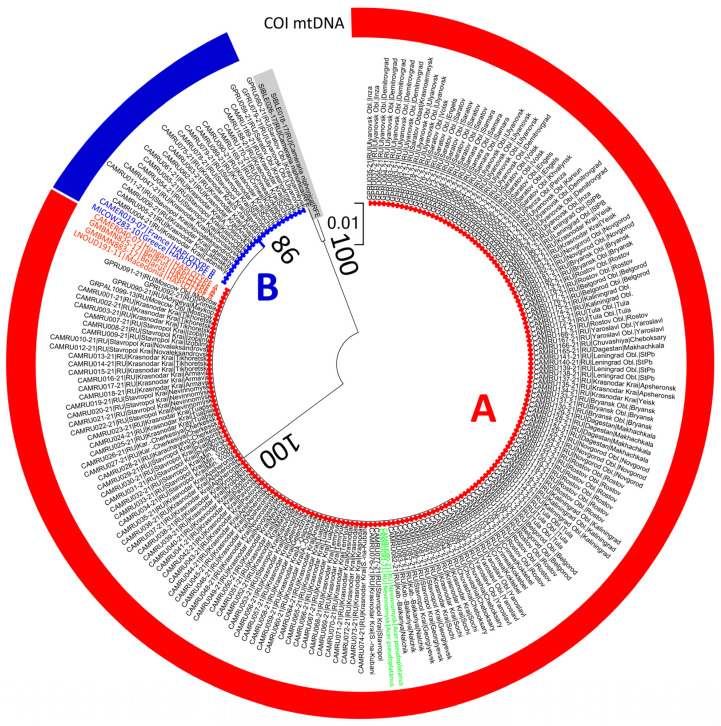
COI tree of *Cameraria ohridella* showing two clusters corresponding to haplotypes A (red) and B (blue) in the European part of Russia. The sequences borrowed from BOLD, corresponding to haplotypes A and B, are indicated in red and blue, respectively; two sequences of *C. ohridella* from *Acer pseudoplatanus* are colored in green; all other sequences originate from *Aesculus hippocastanum*. An outgroup, i.e., two specimens of *C. niphonica* from the Russian Far East (RFE) used to root the tree, is shaded in gray. Each specimen is indicated by the BOLD process ID (begins with CAMRU etc.), followed by a country, region, and locality. Bootstrap values are given next to the corresponding branches.

On the phylogenetic COI tree, the sequences of *C. ohridella* from the European part of Russia, together with six sequences borrowed from BOLD (from Belarus, France, Greece, Hungary, and Macedonia), formed two clusters with significant statistical support (Figure 2). 

In the European part of Russia, haplotype A was detected in 179 out of 201 specimens of *C. ohridella* (i.e., 89%), including in two larvae of *C. ohridella* dissected from leaf mines on *Acer pseudoplatanus* (Figure 2). Haplotype B was found in 22 specimens (11%) only (Figure 2).

The DNA barcodes of *C. ohridella* from the European part of Russia, assigned to haplotypes A and B, corresponded to the respective sequences from European countries. The specimens from European Russia with haplotype A were identical to those from Hungary (Process ID: CAMER040-07), Belarus (GMBMN825-17, GMBMN883-17) and Macedonia (LNOUD191-11). The specimens from Russia with haplotype B were identical to those from France (CAMER019-07) and Greece (MICOW283-10).

In the studied fragment of the mtDNA COI gene, the presence of two diagnostic mutations was determined, allowing for a reliable divide between the two clusters. In the nucleotide position 181 (3′–5′), guanine was present in haplotype A vs. adenine in haplotype B; in the position 439, thymine was present in haplotype A vs. cytosine in haplotype B.

**Figure 3:**
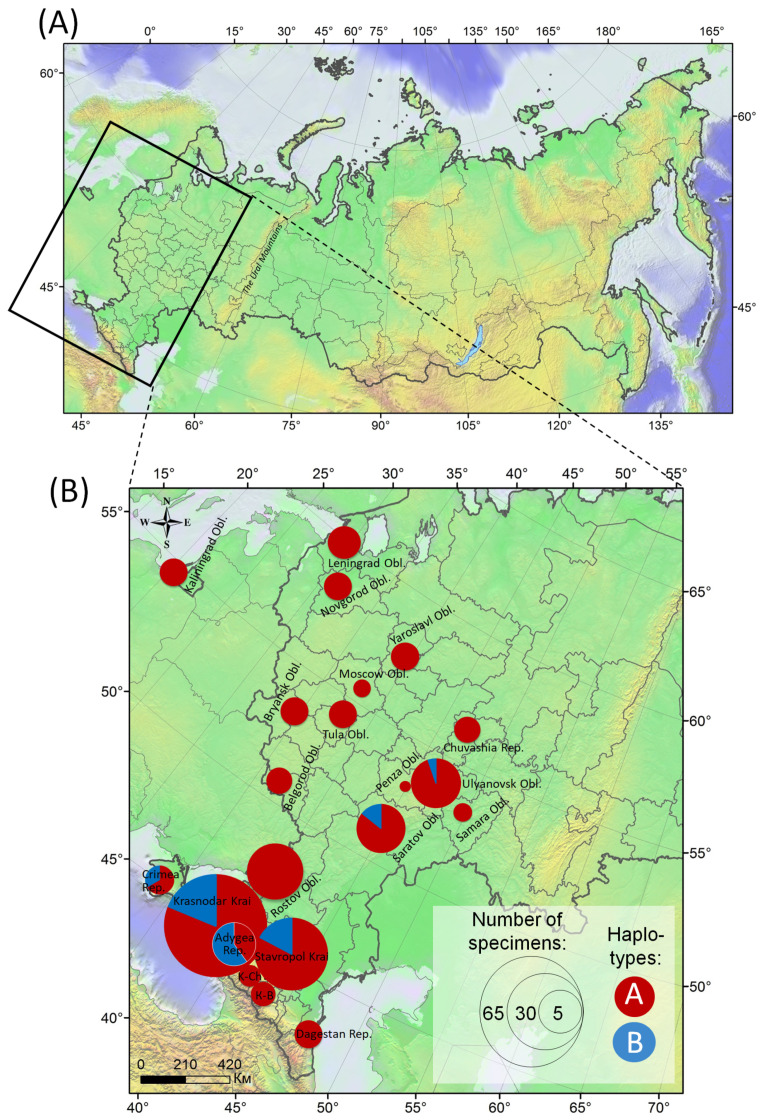
The presence of haplotypes A and B in the populations of *Cameraria ohridella* in the European part of Russia. (**A**) The study area (indicated by the rectangle); (**B**) the haplotypes’ distribution. The pie charts correspond to collected localities. The size of the pie chart reflects the number of sequenced specimens (larvae, pupae, or adults of *C. ohridella*); colored sectors on the diagrams correspond to different haplotypes (see the legend). Abbreviations: K-Ch—Karachay-Cherkessia, K-B—Kabardino-Balkaria. The map is built based on an altitudinal background in ArcGIS 9.3 [45].

Haplotype A was found in almost all studied localities in the European part of Russia and largely dominated across this part of the country (Figure 3). Haplotype B was identified only in populations of the moth in the south of Russia, i.e., in Krasnodar Krai (12 sequences), Stavropol Krai (5), Crimea (2), and about 1000 km to the east on the present limit of the moth distribution in the country, i.e., in the Volga region, in particular, in Saratov (2) and Ulyanovsk (1) Oblasts (Figure 3B). 

In the northern, western, and central regions of Russia, haplotype B was not identified in the studied specimens. Instead, only haplotype A was detected in these parts of the country.

### 3.3. Trophic Associations of C. ohridella with Sapindaceae in Southern Russia

**Figure 4:**
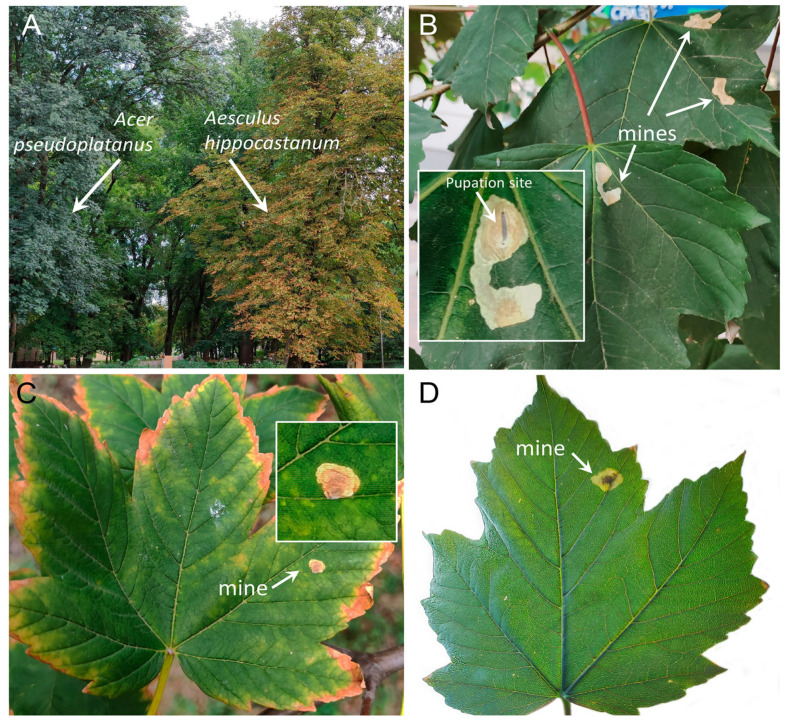
The mines of *Cameraria ohridella* on the leaves of *Acer pseudoplatanus* growing in the vicinity of the horse chestnut *Aesculus hippocastanum* in the Stavropol Krai: in Novoaleksandrovsk (**A**,**B**,**D**) and Nevinnomyssk (**C**), 9–10 August 2021. The insertions in the figures (**B**,**C**) show the mines under magnification. One mine (see insertion in (**B**)) contained live pupa of *C. ohridella*. Photo by N.I. Kirichenko.

Of the studied maple species, the mines of *C. ohridella* were found only on the introduced Western Palearctic maple, *A. pseudoplatanus,* and only in two out of the 30 studied localities in the south of Russia: in the plantations of the cities of Novoaleksandrovsk and Nevinnomyssk (Stavropol Krai) (Figure 4).

In those localities, out of 5000 leaves examined in the lower part of the crowns of 10 trees of *A. pseudoplatanus*, only 18 leaves (0.26% of all examined leaves) carried the pest mines. Overall, 21 mines were found on these leaves: 15 leaves carried 1 mine each, and 3 leaves had 2 mines each. Notably, only maple trees growing in close proximity (up to 5 m distance) to the horse-chestnut trees massively attacked by *C. ohridella* carried the mines (Figure 4). The trees of *A. pseudoplatanus* situated at a distance >100 m from the spot with the infested horse chestnut had no leaf mines of the moth. 

The moth showed the ability to develop through the whole larval stage and pupate feeding on *A. pseudoplatanus* (Figure 4B). In the other maple species examined in Krasnodar and Stavropol Krais, namely, *A. platanoides, A. pictum*, *A. ginnala*, *A. palmatum*, and *A. negundo*, no single mine of *C. ohridella* was detected despite significant search efforts. 

In southern Russia, from 9 to 11 August 2021, the levels of damage of horse-chestnut trees varied in the studied localities (Table 1). 

**Figure 5:**
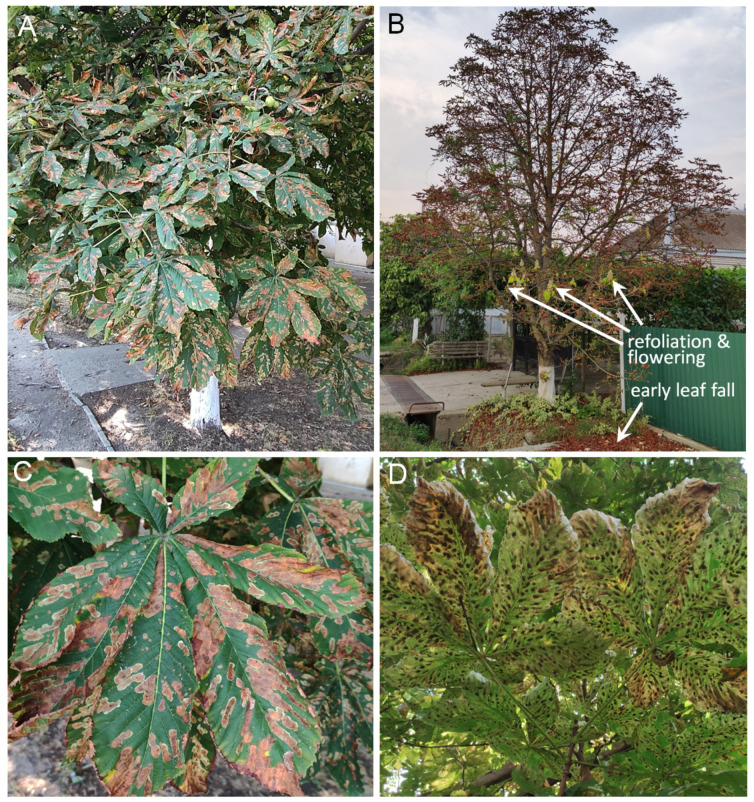
Severely damaged leaves of the horse chestnut *Aesculus hippocastanum* by *Cameraria ohridella* in Southern Russia, Krasnodar Krai, 9 August 2021. Leaves with numerous mines in Anapa (**A**,**C**,**D**); premature leaf fall with subsequent refoliation and flowering of a tree in Slavyansk-na-Kubani (**B**). Photo by N.I. Kirichenko.

In 24 out of 30 localities (80% of all examined localities in the south), the trees of *Ae. hippocastanum* were damaged significantly (i.e., >50 and >75% of leaves carried mines), signaling the outbreak of population densities of *C. ohridella* in the south of the country (Table 1, Figure 5). In four localities (i.e., 13% of all examined localities in the south), the damage caused by *C. ohridella* to *Ae. hippocastanum* trees was moderate (i.e., 25–49% leaves carried the mines), whereas low damage (less than 25% of leaves with mines) was documented only in two localities, in Khadyzhensk (Krasnodar Krai) and Cherkessk (the Karachay-Cherkessia Republic) (Table 1).

The infested horse-chestnut trees were always present in urbanized areas in the studied localities. Nevertheless, in tree belts with the inclusion of *Ae. hippocastanum* along intercity roads both in Krasnodar and Stavropol Krais, the mines of *C. ohridella* were also spotted. Thus, the pest inhibited all surveyed man-made plantings in southern Russia. 

In Slavyansk-na-Kubani (Krasnodar Krai), premature leaf fall with fresh leaves’ regrowth and flowering of the horse chestnuts was documented in early August (Figure 5B). The leaves were severely damaged by *C. ohridella* and infected by the fungus *Guignardia aesculi* (Peck) V. B. Stewart (Ascomycota: Botryosphaeriaceae) (Figure 5B).

## 4. Discussion

The involvement of citizen science in the monitoring of alien species was shown to be beneficial in many ways [35,36,37,46]. Among these are the effective detection and fast reporting on the occurrence of alien pests, the obtainment of specimens for morphological and molecular genetic studies, as well as the saving of time and travel expenditures. The volunteers’ input becomes especially crucial when it is needed to verify the presence of an alien species and collect the specimens on a large scale [47]. This was the case in our study run in European Russia, which has an area of 3.5 mln km² (i.e., about 22% of the whole of Russia and about 35% of Europe). The monitoring and extensive sampling of alien horse-chestnut leaf miners within one field season in a vast area of European Russia became only possible through the involvement of citizen science. Thanks to the volunteers, this invasive alien species was confirmed in 24 administrative regions of European Russia.

The extensive sampling of *C. ohridella* performed by volunteers and our team across European Russia allowed for clarifying the pest invasion pattern and comparing it with that in European countries [25,26,27]. The discovery of only two haplotypes in the horse-chestnut leaf miner populations in European Russia was not surprising. Despite the fact that in the native species populations (the Balkans), a total of 44 haplotypes were identified (both through fresh samplings and the exploration of archival herbaria), in the secondary range of the species, covering most of Europe, only two haplotypes (A and B) have been detected [25,26]. Exactly these two haplotypes were documented in our study in European Russia, with the absolute domination of haplotype A and its presence in all studied localities, whereas haplotype B occurred rarely. With an increase in sampling efforts, it is possible that haplotype B could be found in more studied regions. However, its presence in invasive populations of *C. ohridella* in European Russia could be low and, thus, not comparable with the frequency of occurrence of haplotype A.

High haplotype diversity in the natural range and low haplotype diversity in the secondary range suggest a rapid spread of *C. ohridella* in European countries and European Russia. Only 2 out of 44 known haplotypes of *C. ohridella* (8% of all known haplotypes) were able to spread and effectively settle in new regions. The bottleneck hypothesis, which states that during an invasion process, only a few haplotypes may have an impetus to propagate, whereas the majority of haplotypes (having no such features) remain “locked” in their primary range [48,49], can explain low haplotype diversity in the invasive populations of *C. ohridella* in European Russia.

We believe that the horse-chestnut leaf miner invaded Russia from the neighboring East European countries, where it was documented at the beginning of the 21st century [50,51,52]. The species can spread by different means [28]. Adult moths can spread over long distances with wind currents, like “air plankton”; adults and infected leaves can travel for long distances as “hitchhikers” in/on cars, trucks, and other vehicles; finally, the insects can be imported with host plant seedlings [53,54]. Our observations in southern Russia in 2021 suggest that the moth is able to find its host plant, even a single tree, located 50 km or more away from an infested horse chestnut planted in an urban area.

Taking into account the chronology of *C. ohridella* documented in the regions of European Russia [11,29], we suggest that the species was repeatedly introduced into the country. In Russia, the first record of the moth goes back to 2003 in Kaliningrad, an enclaved region on the Baltic Sea [55]. In 2005, the moth was detected in Moscow [32], about 1000 km east of Kaliningrad and about 450 km from the border with Ukraine, Belarus, and the Baltic countries, where, by that time, the pest was already present [50,51,52]. From 2005 to present day, the species was found in some regions of European Russia, where its host plant, *Ae. hippocastanum* (a tree exotic to Russia), is grown as an ornamental. By 2015, *C. ohridella* was recorded in 13 central regions of European Russia [29]. Soon, the species was detected in southern Russia, in particular, Krasnodar Krai [19,33, present paper], where it presently produces spectacular outbreaks in resort areas. The species could have been accidently introduced here from the central part of Russia via transport (given the attractiveness of this region for tourists) or independently brought from Central Europe, from which place the ornamental plants were transported for landscaping the area of Sochi for the 2014 Winter Olympic Games [19]. The horse-chestnut leaf miner has also penetrated into the Crimean Peninsula and Ciscaucasia [56], including the North Caucasus [57]. In the north of European Russia, *C. ohridella* was recorded in Saint Petersburg in 2013 [58]. So far, the Volga region is known as the easternmost territory in Russia invaded by *C. ohridella* [30]. Here, the species was detected in 2018, suggesting its spread from the western regions in European Russia occupied by the alien pest a few years earlier.

**Figure 6:**
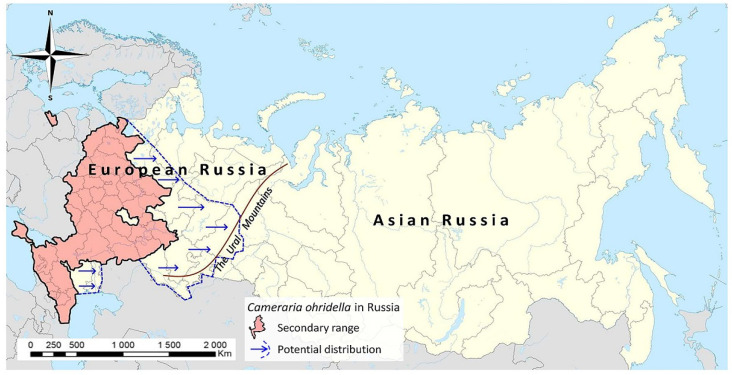
The secondary range of *Cameraria ohridella* in European Russia (shaded in red) based on literature data and observations made by volunteers and our team in 2021. The blue line indicates the approximate boundary that the species can potentially reach by spreading eastwards (taking into account the presence of *Aesculus hippocastanum*, the moth’s host plant, in the regions). The map is built based on an image from © Google, 2022.

The modern distribution map indicates that the pest has already occupied a significant territory of European Russia (Figure 6). Taking into account the number of regions in which *C. ohridella* was confirmed in our study and previously published data [11,29], the species is present in 41 out of 58 (i.e., 70.7%) administrative regions of European Russia, mainly in its western, central, and southern parts. Altogether, these regions occupy the territory of 1.74 mln km^2^, i.e., around 50% of the whole of European Russia. Bearing in mind that the first record of *C. ohridella* in Moscow is dated at 2005, this alien pest has occupied most of the territory of European Russia within only 16 years (up to 2021 inclusive).

Considering that the horse chestnut is used for ornamental purposes in many regions of European Russia, in our opinion, the distribution of *C. ohridella* up to the Ural Mountains can be expected (Figure 6). Further spread of the moth over the Urals is unlikely, because *Ae. hippocastanum* is hardly planted in Western Siberia due to the harsh climate.

Early records in Moscow [59] and European countries [60,61] confirm the ability of *C. ohridella* to switch from *Ae. hippocastanum* to *A. pseudoplatanus*; the moth can also mine the leaves of *A. platanoides* [62]. Maple is not a typical host of the moth and can be occasionally populated if present in spots with heavily infested horse-chestnut trees [28,60]. Indeed, our observations in Southern Russia showed that the moth is able to complete the larval stage and pupate in the leaves of *A. pseudoplatanus*, and it only attacks maple trees growing next to heavily infested horse chestnuts.

The maple (*Acer*) and horse chestnut (*Aesculus*) are phylogenetically close genera belonging to the same subfamily, Hippocastanoideae, of the family Sapindaceae, in which overall, four subfamilies and about 150 genera are known [63]. It remains unclear whether or not *C. ohridella* can colonize maple species originating from other geographic regions, such as East Asia and North America. The maples originating from these regions are used in landscaping in European Russia and may serve as potential hosts for the pest. Exploring the ability of the pest to form new trophic associations would help to determine whether there is a risk that *C. ohridella* can expand its range into East Asia or North America, the motherland of highly ornamental and economically important maples and some *Aesculus* species.

## 5. Conclusions

Our study is a good example of the involvement of citizens in the exploration of a modern range of an invasive horse-chestnut leaf miner in Russia. Thanks to the volunteers, from June to August 2021, *C. ohridella* was documented in 24 administrative regions of the European part of Russia, and the subsequent sampling of the insect specimens was performed for the molecular genetic study.

The DNA barcoding of *C. ohridella* specimens suggests its rapid invasion in the European part of Russia, corresponding to the phylogeographic pattern described for this invasive species in Eastern and Western Europe. Only two haplotypes (A and B), known by their “invasive” character in European countries, were recorded in the European part of Russia. Taking into account the chronology of the species’ detection in Russia, the introduction of the pest could have happened repeatedly, most likely from East European countries, and its further spread into Russia could be the result of an unintentional introduction and natural spread from the infected domestic regions. To date, the moth has spread into the territory of the European part of Russia, up to and inclusive of the Volga region. Bearing in mind that the horse chestnut, *Ae. hippocastanum*, a host plant of *C. ohridella*, is present in urban ecosystems in many regions of the European part of Russia, further spread of the pest up to the Ural Mountains could not be ruled out. 

The host shift in *C. ohridella,* in particular the ability of the moth to form a trophic association with sycamore, *A. pseudoplatanus,* might allow the pest to continue spreading, as well as support the populations during heavy outbreaks in *Ae. hippocastanum*.

As a prospect for future research, it would be important to explore the ability of the horse-chestnut leaf miner to switch to other species of horse chestnuts (*Aesculus* spp.) and maples (*Acer* spp.) present in the modern range of the pest in the European continent and naturally growing in East Asia and North America.

## Figures and Tables

**Table 1:** Damage to the horse chestnut trees caused by the horse-chestnut leaf miner in Southern Russia, 9–11 August 2021.

No.	Locality (City, Village)	Damage Level *			
		Low 1–25%	Moderate 26–50%	High 51–75%	Severe >75%
Krasnodar Krai					
1	Anapa				
2	Apsheronsk				
3	Armavir				
4	Arkhipo-Osipovka				
5	Gelendzhik				
6	Krasnodar				
7	Kropotkin				
8	Labinsk				
9	Novomikhailovsky				
10	Novorossiysk				
11	Slavyansk-na-Kubani				
12	Sochi, Adler district				
13	Sochi, Central district				
14	Stanitsa Pavlovskaya				
15	Temryuk				
16	Tikhoretsk				
17	Tuapse				
18	Khadyzhensk				
19	Khopersky				
The Republic of Adygea					
20	Maykop				
Stavropol Krai					
21	Izobilny				
22	Novoaleksandrovsk				
23	Nevinnomyssk				
24	Pyatigorsk				
25	Mineralnye Vody				
26	Kislovodsk				
27	Stavropol				
28	Georgievsk				
The Republic of Kabardino-Balkaria					
29	Nalchik				
The Republic of Karachay-Cherkessia					
30	Cherkessk				
	TOTAL number of localities by the damage level	2 (7 %)	4 (13 %)	10 (33 %)	14 (47 %)

* According to the assessment of a relative number of leaves with mines in the lower part of tree crown (see the paragraph 2.5. Trophic Associations). Different colors reflect different damage levels: light gray—low, dark gray—moderate, blackish—high, red—severe.

## Data Availability

The genetic data used in the study are publicly accessible in BOLD using the link: dx.doi.org/10.5883/DS-CAMRUS (accessed on 24 January 2023).

## Supplementary Materials

The following supporting information can be downloaded at: https://www.mdpi.com/article/10.3390/insects14020117/s1, Table S1: The specimens of *Cameraria ohridella* from the European part of Russia involved into molecular genetic analysis and the species’ sequences borrowed from BOLD for comparison. For each specimen, sample ID and process ID are provided, linking the records in the BOLD database with the voucher specimen data.
