# Supplementary material for: Invasion Genetics of the Horse-Chestnut Leaf Miner, Cameraria ohridella (Lepidoptera: Gracillariidae), in European Russia: A Case of Successful Involvement of Citizen Science in Studying an Alien Insect Pest
Source: Insects. 2023 Jan 24;14(2):117. doi: 10.3390/insects14020117 (PMC9961473; doi:10.3390/insects14020117)
Supplement: Supplementary file 1 [file insects-14-00117-s001.zip › insects-2144072-supplementary.pdf]

The Invasion Genetics of the Horse-Chestnut Leaf Miner, *Cameraria ohridella* (Lepidoptera: Gracillariidae), in European Russia: the Case of Successful Involvement of Citizen Science in Studying an Alien Insect Pest

AUTHORS: Natalia I. Kirichenko, Natalia N. Karpun, Elena N. Zhuravleva, Elena I. Shoshina, Vasily V. Anikin, Dmitrii L. Musolin

Table S1. The specimens of *Cameraria ohridella* from the European part of Russia involved into molecular genetic analysis and the species sequences borrowed from BOLD for comparison. For each specimen, sample ID and process ID are provided, linking the records in the BOLD database with the voucher specimen data.

No.	Sample ID	Process ID	Country	Region	Place	Collection data	Collector	GenBank Accession
**Specimens sequenced in the present study**								
1.	NK-20-41	GPRU090-21	Russia	Adygeya Rep.	Maykop	25-Jun-2021	Demidko D.	MZ329804
2.	NK1024	CAMRU109-21	Russia	Belgorod Oblast	Belgorod	18-Jul-2021	Stuchaev V.	OQ079082
3.	NK1025	CAMRU110-21	Russia	Belgorod Oblast	Belgorod	18-Jul-2021	Stuchaev V.	OQ079006
4.	NK1091	CAMRU176-21	Russia	Belgorod Oblast	Belgorod	18-Jul-2021	Stuchaev V.	OQ078930
5.	NK1092	CAMRU177-21	Russia	Belgorod Oblast	Belgorod	18-Jul-2021	Stuchaev V.	OQ078947
6.	NK1045	CAMRU130-21	Russia	Bryansk Oblast	Bryansk	11-Jul-2021	unknown	OQ079069
7.	NK1046	CAMRU131-21	Russia	Bryansk Oblast	Bryansk	11-Jul-2021	unknown	OQ078950
8.	NK1048	CAMRU133-21	Russia	Bryansk Oblast	Bryansk	11-Jul-2021	unknown	OQ079037
9.	NK1095	CAMRU180-21	Russia	Bryansk Oblast	Bryansk	11-Jul-2021	unknown	OQ078997
10.	NK1096	CAMRU181-21	Russia	Bryansk Oblast	Bryansk	11-Jul-2021	unknown	OQ078955
11.	NK1011	CAMRU096-21	Russia	Chuvashia Rep.	Cheboksary	03-Aug-2021	Smirnova N.	OQ078954
12.	NK1012	CAMRU097-21	Russia	Chuvashia Rep.	Cheboksary	03-Aug-2021	Smirnova N.	OQ078953
13.	NK1013	CAMRU098-21	Russia	Chuvashia Rep.	Cheboksary	03-Aug-2021	Smirnova N.	OQ078932
14.	NK1081	CAMRU166-21	Russia	Chuvashia Rep.	Cheboksary	03-Aug-2021	Smirnova N.	OQ079031
15.	NK1017	CAMRU102-21	Russia	Crimea Rep.	Koktebel	13-Jul-2021	Smirnova N.	OQ079064
16.	NK1018	CAMRU103-21	Russia	Crimea Rep.	Koktebel	13-Jul-2021	Smirnova N.	OQ078958
17.	NK1019	CAMRU104-21	Russia	Crimea Rep.	Koktebel	13-Jul-2021	Smirnova N.	OQ079046

No.	Sample ID	Process ID	Country	Region	Place	Collection data	Collector	GenBank Accession
18.	NK1084	CAMRU169-21	Russia	Crimea Rep.	Koktebel	13-Jul-2021	Smirnova N.	OQ079100
19.	NK1085	CAMRU170-21	Russia	Crimea Rep.	Koktebel	13-Jul-2021	Smirnova N.	OQ079049
20.	NK-20-43	GPRU092-21	Russia	Crimea Rep.	Feodosia	20-Aug-2017	Akulov E.	MZ329805
21.	NK1041	CAMRU126-21	Russia	Dagestan Rep.	Makhachkala	07-Jul-2021	Ilina E.	OQ079016
22.	NK1042	CAMRU127-21	Russia	Dagestan Rep.	Makhachkala	07-Jul-2021	Ilina E.	OQ079027
23.	NK1043	CAMRU128-21	Russia	Dagestan Rep.	Makhachkala	07-Jul-2021	Ilina E.	OQ078981
24.	NK1044	CAMRU129-21	Russia	Dagestan Rep.	Makhachkala	07-Jul-2021	Ilina E.	OQ079094
25.	NK1080	CAMRU165-21	Russia	Dagestan Rep.	Makhachkala	07-Jul-2021	Ilina E.	OQ079097
26.	NK997	CAMRU082-21	Russia	Kabardino-Balkaria Rep.	Nalchik	08-Jun-2021	Akulov E.	OQ078943
27.	NK998	CAMRU083-21	Russia	Kabardino-Balkaria Rep.	Nalchik	08-Jun-2021	Akulov E.	OQ079063
28.	NK999	CAMRU084-21	Russia	Kabardino-Balkaria Rep.	Nalchik	08-Jun-2021	Akulov E.	OQ078921
29.	NK1029	CAMRU114-21	Russia	Kaliningrad Oblast	Kaliningrad	17-Jul-2021	Didorenko V.	OQ079071
30.	NK1030	CAMRU115-21	Russia	Kaliningrad Oblast	Kaliningrad	17-Jul-2021	Didorenko V.	OQ079017
31.	NK1031	CAMRU116-21	Russia	Kaliningrad Oblast	Kaliningrad	17-Jul-2021	Didorenko V.	OQ079058
32.	NK1089	CAMRU174-21	Russia	Kaliningrad Oblast	Kaliningrad	17-Jul-2021	Didorenko V.	OQ079033
33.	NK1090	CAMRU175-21	Russia	Kaliningrad Oblast	Kaliningrad	17-Jul-2021	Didorenko V.	OQ079095
34.	NK941	CAMRU026-21	Russia	Karachay-Cherkessia Rep.	Cherkessk	10-Aug-2021	Kirichenko N., Karpun N.	OQ079045
35.	NK942	CAMRU027-21	Russia	Karachay-Cherkessia Rep.	Cherkessk	10-Aug-2021	Kirichenko N., Karpun N.	OQ078991
36.	NK943	CAMRU028-21	Russia	Karachay-Cherkessia Rep.	Cherkessk	10-Aug-2021	Kirichenko N., Karpun N.	OQ078972
37.	NK977	CAMRU062-21	Russia	Krasnodar Krai	Anapa	09-Aug-2021	Kirichenko N., Karpun N.	OQ079074
38.	NK978	CAMRU063-21	Russia	Krasnodar Krai	Anapa	09-Aug-2021	Kirichenko N., Karpun N.	OQ078995
39.	NK979	CAMRU064-21	Russia	Krasnodar Krai	Anapa	09-Aug-2021	Kirichenko N., Karpun N.	OQ078977
40.	NK950	CAMRU035-21	Russia	Krasnodar Krai	Apsheronsk	11-Aug-2021	Kirichenko N., Karpun N.	OQ079042
41.	NK951	CAMRU036-21	Russia	Krasnodar Krai	Apsheronsk	11-Aug-2021	Kirichenko N., Karpun N.	OQ079068

No.	Sample ID	Process ID	Country	Region	Place	Collection data	Collector	GenBank Accession
42.	NK952	CAMRU037-21	Russia	Krasnodar Krai	Apsheronsk	11-Aug-2021	Kirichenko N., Karpun N.	OQ079043
43.	NK974	CAMRU059-21	Russia	Krasnodar Krai	Arkhipo-Osipovka	09-Aug-2021	Kirichenko N., Karpun N.	OQ078983
44.	NK975	CAMRU060-21	Russia	Krasnodar Krai	Arkhipo-Osipovka	09-Aug-2021	Kirichenko N., Karpun N.	OQ079085
45.	NK976	CAMRU061-21	Russia	Krasnodar Krai	Arkhipo-Osipovka	09-Aug-2021	Kirichenko N., Karpun N.	OQ079010
46.	NK931	CAMRU016-21	Russia	Krasnodar Krai	Armavir	11-Aug-2021	Kirichenko N., Karpun N.	OQ078993
47.	NK932	CAMRU017-21	Russia	Krasnodar Krai	Armavir	11-Aug-2021	Kirichenko N., Karpun N.	OQ079055
48.	NK933	CAMRU018-21	Russia	Krasnodar Krai	Armavir	11-Aug-2021	Kirichenko N., Karpun N.	OQ079078
49.	NK971	CAMRU056-21	Russia	Krasnodar Krai	Gelendzhik	09-Aug-2021	Kirichenko N., Karpun N.	OQ079047
50.	NK972	CAMRU057-21	Russia	Krasnodar Krai	Gelendzhik	09-Aug-2021	Kirichenko N., Karpun N.	OQ078985
51.	NK973	CAMRU058-21	Russia	Krasnodar Krai	Gelendzhik	09-Aug-2021	Kirichenko N., Karpun N.	OQ078963
52.	NK959	CAMRU044-21	Russia	Krasnodar Krai	Khadyzhensk	11-Aug-2021	Kirichenko N., Karpun N.	OQ079088
53.	NK960	CAMRU045-21	Russia	Krasnodar Krai	Khadyzhensk	11-Aug-2021	Kirichenko N., Karpun N.	OQ078920
54.	NK961	CAMRU046-21	Russia	Krasnodar Krai	Khadyzhensk	11-Aug-2021	Kirichenko N., Karpun N.	OQ079039
55.	NK916	CAMRU001-21	Russia	Krasnodar Krai	Khopersky	10-Aug-2021	Kirichenko N., Karpun N.	OQ078980
56.	NK917	CAMRU002-21	Russia	Krasnodar Krai	Khopersky	10-Aug-2021	Kirichenko N., Karpun N.	OQ078969
57.	NK918	CAMRU003-21	Russia	Krasnodar Krai	Khopersky	10-Aug-2021	Kirichenko N., Karpun N.	OQ079104
58.	NK986	CAMRU071-21	Russia	Krasnodar Krai	Krasnodar	09-Aug-2021	Kirichenko N., Karpun N.	OQ079025
59.	NK987	CAMRU072-21	Russia	Krasnodar Krai	Krasnodar	09-Aug-2021	Kirichenko N., Karpun N.	OQ078966
60.	NK988	CAMRU073-21	Russia	Krasnodar Krai	Krasnodar	09-Aug-2021	Kirichenko N., Karpun N.	OQ078990
61.	NK953	CAMRU038-21	Russia	Krasnodar Krai	Kropotkin	10-Aug-2021	Kirichenko N., Karpun N.	OQ079023
62.	NK954	CAMRU039-21	Russia	Krasnodar Krai	Kropotkin	10-Aug-2021	Kirichenko N., Karpun N.	OQ078919
63.	NK955	CAMRU040-21	Russia	Krasnodar Krai	Kropotkin	10-Aug-2021	Kirichenko N., Karpun N.	OQ078933
64.	NK919	CAMRU004-21	Russia	Krasnodar Krai	Labinsk	11-Aug-2021	Kirichenko N., Karpun N.	OQ078942
65.	NK920	CAMRU005-21	Russia	Krasnodar Krai	Labinsk	11-Aug-2021	Kirichenko N., Karpun N.	OQ079081
66.	NK921	CAMRU006-21	Russia	Krasnodar Krai	Labinsk	11-Aug-2021	Kirichenko N., Karpun N.	OQ079075
67.	NK965	CAMRU050-21	Russia	Krasnodar Krai	Maykop	11-Aug-2021	Kirichenko N., Karpun N.	OQ079089
68.	NK966	CAMRU051-21	Russia	Krasnodar Krai	Maykop	11-Aug-2021	Kirichenko N., Karpun N.	OQ078948
69.	NK967	CAMRU052-21	Russia	Krasnodar Krai	Maykop	11-Aug-2021	Kirichenko N., Karpun N.	OQ078927

No.	Sample ID	Process ID	Country	Region	Place	Collection data	Collector	GenBank Accession
70.	NK938	CAMRU023-21	Russia	Krasnodar Krai	Novomikhailovsky	09-Aug-2021	Kirichenko N., Karpun N.	OQ079040
71.	NK939	CAMRU024-21	Russia	Krasnodar Krai	Novomikhailovsky	09-Aug-2021	Kirichenko N., Karpun N.	OQ079044
72.	NK940	CAMRU025-21	Russia	Krasnodar Krai	Novomikhailovsky	09-Aug-2021	Kirichenko N., Karpun N.	OQ079066
73.	NK962	CAMRU047-21	Russia	Krasnodar Krai	Novorossiysk	09-Aug-2021	Kirichenko N., Karpun N.	OQ079000
74.	NK963	CAMRU048-21	Russia	Krasnodar Krai	Novorossiysk	09-Aug-2021	Kirichenko N., Karpun N.	OQ078914
75.	NK964	CAMRU049-21	Russia	Krasnodar Krai	Novorossiysk	09-Aug-2021	Kirichenko N., Karpun N.	OQ079083
76.	NK989	CAMRU074-21	Russia	Krasnodar Krai	Slavyansk-na-Kubani	09-Aug-2021	Kirichenko N., Karpun N.	OQ078937
77.	NK990	CAMRU075-21	Russia	Krasnodar Krai	Slavyansk-na-Kubani	09-Aug-2021	Kirichenko N., Karpun N.	OQ078916
78.	NK991	CAMRU076-21	Russia	Krasnodar Krai	Slavyansk-na-Kubani	09-Aug-2021	Kirichenko N., Karpun N.	OQ079029
79.	NK1103	CAMRU188-21	Russia	Krasnodar Krai	Sochi	21-Jun-2021	Zhuravleva E., Shoshina E.	OQ078998
80.	NK1104	CAMRU189-21	Russia	Krasnodar Krai	Sochi	21-Jun-2021	Zhuravleva E., Shoshina E.	OQ078999
81.	NK1007	CAMRU092-21	Russia	Krasnodar Krai	Sochi, Adlersky district	21-Jun-2021	Zhuravleva E., Shoshina E.	OQ078965
82.	NK1008	CAMRU093-21	Russia	Krasnodar Krai	Sochi, Adlersky district	21-Jun-2021	Zhuravleva E., Shoshina E.	OQ079070
83.	NK1009	CAMRU094-21	Russia	Krasnodar Krai	Sochi, Adlersky district	21-Jun-2021	Zhuravleva E., Shoshina E.	OQ078934
84.	NK1004	CAMRU089-21	Russia	Krasnodar Krai	Sochi, Central district	21-Jun-2021	Zhuravleva E., Shoshina E.	OQ078952
85.	NK1005	CAMRU090-21	Russia	Krasnodar Krai	Sochi, Central district	21-Jun-2021	Zhuravleva E., Shoshina E.	OQ079084
86.	NK1006	CAMRU091-21	Russia	Krasnodar Krai	Sochi, Central district	21-Jun-2021	Zhuravleva E., Shoshina E.	OQ078940
87.	NK956	CAMRU041-21	Russia	Krasnodar Krai	Stanitsa Pavlovskaya	10-Aug-2021	Kirichenko N., Karpun N.	OQ079053
88.	NK957	CAMRU042-21	Russia	Krasnodar Krai	Stanitsa Pavlovskaya	10-Aug-2021	Kirichenko N., Karpun N.	OQ078984

No.	Sample ID	Process ID	Country	Region	Place	Collection data	Collector	GenBank Accession
89.	NK958	CAMRU043-21	Russia	Krasnodar Krai	Stanitsa Pavlovskaya	10-Aug-2021	Kirichenko N., Karpun N.	OQ078964
90.	NK983	CAMRU068-21	Russia	Krasnodar Krai	Temryuk	09-Aug-2021	Kirichenko N., Karpun N.	OQ079077
91.	NK984	CAMRU069-21	Russia	Krasnodar Krai	Temryuk	09-Aug-2021	Kirichenko N., Karpun N.	OQ079101
92.	NK985	CAMRU070-21	Russia	Krasnodar Krai	Temryuk	09-Aug-2021	Kirichenko N., Karpun N.	OQ079019
93.	NK928	CAMRU013-21	Russia	Krasnodar Krai	Tihkoretsk	10-Aug-2021	Kirichenko N., Karpun N.	OQ079007
94.	NK929	CAMRU014-21	Russia	Krasnodar Krai	Tihkoretsk	10-Aug-2021	Kirichenko N., Karpun N.	OQ079102
95.	NK930	CAMRU015-21	Russia	Krasnodar Krai	Tihkoretsk	10-Aug-2021	Kirichenko N., Karpun N.	OQ078925
96.	NK980	CAMRU065-21	Russia	Krasnodar Krai	Tuapse	09-Aug-2021	Kirichenko N., Karpun N.	OQ079105
97.	NK981	CAMRU066-21	Russia	Krasnodar Krai	Tuapse	09-Aug-2021	Kirichenko N., Karpun N.	OQ079001
98.	NK982	CAMRU067-21	Russia	Krasnodar Krai	Tuapse	09-Aug-2021	Kirichenko N., Karpun N.	OQ078917
99.	NK1049	CAMRU134-21	Russia	Krasnodar Krai	Yeisk	30-Jul-2021	Shestakova M.	OQ078971
100.	NK1050	CAMRU135-21	Russia	Krasnodar Krai	Yeisk	30-Jul-2021	Shestakova M.	OQ079080
101.	NK1052	CAMRU137-21	Russia	Krasnodar Krai	Yeisk	30-Jul-2021	Shestakova M.	OQ079093
102.	NK1099	CAMRU184-21	Russia	Krasnodar Krai	Yeisk	30-Jul-2021	Shestakova M.	OQ079060
103.	NK1100	CAMRU185-21	Russia	Krasnodar Krai	Yeisk	30-Jul-2021	Shestakova M.	OQ079106
104.	NK1053	CAMRU138-21	Russia	Leningrad Oblast	Saint Petersburg	10-Jul-2021	Margirova M.	OQ079011
105.	NK1054	CAMRU139-21	Russia	Leningrad Oblast	Saint Petersburg	10-Jul-2021	Margirova M.	OQ079038
106.	NK1055	CAMRU140-21	Russia	Leningrad Oblast	Saint Petersburg	09-Jul-2021	Margirova M.	OQ079028
107.	NK1056	CAMRU141-21	Russia	Leningrad Oblast	Saint Petersburg	09-Jul-2021	Margirova M.	OQ078913
108.	NK1101	CAMRU186-21	Russia	Leningrad Oblast	Saint Petersburg	12-Jul-2021	Margirova M.	OQ079096
109.	NK1102	CAMRU187-21	Russia	Leningrad Oblast	Saint Petersburg	12-Jul-2021	Margirova M.	OQ078978
110.	NK-20-42	GPRU091-21	Russia	Moscow Oblast	Moscow suburb	20-Sep-2021	Agafonova J.	MZ329803
111.	NK55	GRPAL1099-13	Russia	Moscow Oblast	Moscow suburb	21-Jun-2010	Kirichenko N.	MW213908
112.	NK1038	CAMRU123-21	Russia	Novgorod Oblast	Veliky Novgorod	09-Jul-2021	Timoshina O.	OQ078938
113.	NK1039	CAMRU124-21	Russia	Novgorod Oblast	Veliky Novgorod	09-Jul-2021	Timoshina O.	OQ078994
114.	NK1040	CAMRU125-21	Russia	Novgorod Oblast	Veliky Novgorod	09-Jul-2021	Timoshina O.	OQ079079
115.	NK1097	CAMRU182-21	Russia	Novgorod Oblast	Veliky Novgorod	09-Jul-2021	Timoshina O.	OQ079086

No.	Sample ID	Process ID	Country	Region	Place	Collection data	Collector	GenBank Accession
116.	NK1098	CAMRU183-21	Russia	Novgorod Oblast	Veliky Novgorod	09-Jul-2021	Timoshina O.	OQ078987
117.	VA-20-1	GPRU052-21	Russia	Penza Oblast	Penza	26-Sep-2020	Anikin V.V.	OQ079091
118.	NK1020	CAMRU105-21	Russia	Rostov Oblast	Rostov-on-Don	14-Jul-2021	Levchenko I.	OQ078988
119.	NK1021	CAMRU106-21	Russia	Rostov Oblast	Rostov-on-Don	14-Jul-2021	Levchenko I.	OQ078912
120.	NK1022	CAMRU107-21	Russia	Rostov Oblast	Rostov-on-Don	14-Jul-2021	Levchenko I.	OQ078986
121.	NK1032	CAMRU117-21	Russia	Rostov Oblast	Rostov-on-Don	28-May-2021	Bulgakov T.S.	OQ079026
122.	NK1033	CAMRU118-21	Russia	Rostov Oblast	Rostov-on-Don	28-May-2021	Bulgakov T.S.	OQ078929
123.	NK1034	CAMRU119-21	Russia	Rostov Oblast	Rostov-on-Don	28-May-2021	Bulgakov T.S.	OQ079050
124.	NK1086	CAMRU171-21	Russia	Rostov Oblast	Rostov-on-Don	14-Jul-2021	Levchenko I.	OQ078982
125.	NK1035	CAMRU120-21	Russia	Rostov Oblast	Shakhty	19-Jul-2021	Bulgakov T.S.	OQ078924
126.	NK1036	CAMRU121-21	Russia	Rostov Oblast	Shakhty	19-Jul-2021	Bulgakov T.S.	OQ079005
127.	NK1037	CAMRU122-21	Russia	Rostov Oblast	Shakhty	19-Jul-2021	Bulgakov T.S.	OQ079065
128.	NK1093	CAMRU178-21	Russia	Rostov Oblast	Shakhty	20-Jul-2021	Bulgakov T.C.	OQ078959
129.	NK1094	CAMRU179-21	Russia	Rostov Oblast	Shakhty	20-Jul-2021	Bulgakov T.C.	OQ078918
130.	VA-20-13	GPRU064-21	Russia	Samara Oblast	Samara	17-Oct-2020	Anikin V.	OQ078979
131.	VA-20-14	GPRU065-21	Russia	Samara Oblast	Samara	17-Oct-2020	Anikin V.	OQ078970
132.	VA-20-15	GPRU066-21	Russia	Samara Oblast	Samara	17-Oct-2020	Anikin V.	OQ078992
133.	VA-20-3	GPRU054-21	Russia	Saratovsk Oblast	Engels	19-Jul-2020	Anikin V.	OQ078967
134.	VA-20-4	GPRU055-21	Russia	Saratovsk Oblast	Engels	19-Jul-2020	Anikin V.	OQ078951
135.	VA-20-24	GPRU075-21	Russia	Saratovsk Oblast	Engels	19-Jul-2020	Anikin V.	OQ078946
136.	VA-20-2	GPRU053-21	Russia	Saratovsk Oblast	Khvalynsk	19-Oct-2020	Anikin V.	OQ079098
137.	VA-20-8	GPRU059-21	Russia	Saratovsk Oblast	Krasnoarmeysk	28-Jul-2020	Anikin V.	OQ079072
138.	VA-20-29	GPRU080-21	Russia	Saratovsk Oblast	Krasnoarmeysk	28-Jul-2020	Zolotuhin V.	OQ079087
139.	VA-20-30	GPRU081-21	Russia	Saratovsk Oblast	Krasnoarmeysk	28-Jul-2020	Zolotuhin V.	OQ079003
140.	VA-20-16	GPRU067-21	Russia	Saratovsk Oblast	Saratov	15-Jul-2020	Anikin V.	OQ079022
141.	VA-20-17	GPRU068-21	Russia	Saratovsk Oblast	Saratov	14-Jul-2020	Anikin V.	OQ078956
142.	VA-20-21	GPRU072-21	Russia	Saratovsk Oblast	Saratov	24-Jul-2020	Anikin V.	OQ079041
143.	VA-20-22	GPRU073-21	Russia	Saratovsk Oblast	Saratov	16-Sep-2020	Anikin V.	OQ079032

No.	Sample ID	Process ID	Country	Region	Place	Collection data	Collector	GenBank Accession
144.	VA-20-5	GPRU056-21	Russia	Saratovsk Oblast	Volsk	02-Aug-2020	Anikin V.	OQ078976
145.	VA-20-6	GPRU057-21	Russia	Saratovsk Oblast	Volsk	02-Aug-2020	Anikin V.	OQ079035
146.	VA-20-25	GPRU076-21	Russia	Saratovsk Oblast	Volsk	02-Aug-2020	Anikin V.	OQ078962
147.	NK1000	CAMRU085-21	Russia	Stavropol Krai	Georgiyevsk	09-Jun-2021	Kirichenko N., Karpun N.	OQ079015
148.	NK1001	CAMRU086-21	Russia	Stavropol Krai	Georgiyevsk	09-Jun-2021	Kirichenko N., Karpun N.	OQ078926
149.	NK1002	CAMRU087-21	Russia	Stavropol Krai	Georgiyevsk	09-Jun-2021	Kirichenko N., Karpun N.	OQ079030
150.	NK922	CAMRU007-21	Russia	Stavropol Krai	Izobilny	10-Aug-2021	Kirichenko N., Karpun N.	OQ078968
151.	NK923	CAMRU008-21	Russia	Stavropol Krai	Izobilny	10-Aug-2021	Kirichenko N., Karpun N.	OQ079056
152.	NK924	CAMRU009-21	Russia	Stavropol Krai	Izobilny	10-Aug-2021	Kirichenko N., Karpun N.	OQ079073
153.	NK968	CAMRU053-21	Russia	Stavropol Krai	Kislovodsk	11-Aug-2021	Kirichenko N., Karpun N.	OQ079002
154.	NK969	CAMRU054-21	Russia	Stavropol Krai	Kislovodsk	11-Aug-2021	Kirichenko N., Karpun N.	OQ079021
155.	NK970	CAMRU055-21	Russia	Stavropol Krai	Kislovodsk	11-Aug-2021	Kirichenko N., Karpun N.	OQ079108
156.	NK947	CAMRU032-21	Russia	Stavropol Krai	Mineralnye Vody	10-Aug-2021	Kirichenko N., Karpun N.	OQ079062
157.	NK948	CAMRU033-21	Russia	Stavropol Krai	Mineralnye Vody	10-Aug-2021	Kirichenko N., Karpun N.	OQ079009
158.	NK949	CAMRU034-21	Russia	Stavropol Krai	Mineralnye Vody	10-Aug-2021	Kirichenko N., Karpun N.	OQ079107
159.	NK934	CAMRU019-21	Russia	Stavropol Krai	Nevinnomysk	10-Aug-2021	Kirichenko N., Karpun N.	OQ079020
160.	NK935	CAMRU020-21	Russia	Stavropol Krai	Nevinnomysk	10-Aug-2021	Kirichenko N., Karpun N.	OQ078928
161.	NK936	CAMRU021-21	Russia	Stavropol Krai	Nevinnomysk	10-Aug-2021	Kirichenko N., Karpun N.	OQ078949
162.	NK937	CAMRU022-21	Russia	Stavropol Krai	Nevinnomysk	10-Aug-2021	Kirichenko N., Karpun N.	OQ078989
163.	NK995	CAMRU080-21	Russia	Stavropol Krai	Nevinnomysk	10-Aug-2021	Kirichenko N., Karpun N.	OQ079067
164.	NK996	CAMRU081-21	Russia	Stavropol Krai	Nevinnomysk	10-Aug-2021	Kirichenko N., Karpun N.	OQ078915
165.	NK925	CAMRU010-21	Russia	Stavropol Krai	Novoaleksandrovsk	10-Aug-2021	Kirichenko N., Karpun N.	OQ079057
166.	NK926	CAMRU011-21	Russia	Stavropol Krai	Novoaleksandrovsk	10-Aug-2021	Kirichenko N., Karpun N.	OQ078935
167.	NK927	CAMRU012-21	Russia	Stavropol Krai	Novoaleksandrovsk	10-Aug-2021	Kirichenko N., Karpun N.	OQ078961
168.	NK944	CAMRU029-21	Russia	Stavropol Krai	Pyatigorsk	10-Aug-2021	Kirichenko N., Karpun N.	OQ079051
169.	NK945	CAMRU030-21	Russia	Stavropol Krai	Pyatigorsk	10-Aug-2021	Kirichenko N., Karpun N.	OQ079014
170.	NK946	CAMRU031-21	Russia	Stavropol Krai	Pyatigorsk	10-Aug-2021	Kirichenko N., Karpun N.	OQ079103
171.	NK992	CAMRU077-21	Russia	Stavropol Krai	Stavropol	08-Aug-2021	Lemeshko V.	OQ078957

No.	Sample ID	Process ID	Country	Region	Place	Collection data	Collector	GenBank Accession
172.	NK993	CAMRU078-21	Russia	Stavropol Krai	Stavropol	08-Aug-2021	Lemeshko V.	OQ078944
173.	NK994	CAMRU079-21	Russia	Stavropol Krai	Stavropol	08-Aug-2021	Lemeshko V.	OQ079034
174.	NK1026	CAMRU111-21	Russia	Tula Oblast	Tula	24-Jul-2021	Kosheleva S.	OQ078936
175.	NK1027	CAMRU112-21	Russia	Tula Oblast	Tula	24-Jul-2021	Kosheleva S.	OQ079076
176.	NK1028	CAMRU113-21	Russia	Tula Oblast	Tula	24-Jul-2021	Kosheleva S.	OQ078922
177.	NK1087	CAMRU172-21	Russia	Tula Oblast	Tula	24-Jul-2021	Kosheleva S.	OQ079036
178.	NK1088	CAMRU173-21	Russia	Tula Oblast	Tula	24-Jul-2021	Kosheleva S.	OQ079012
179.	NK-20-44	GPRU093-21	Russia	Ulyanovsk Oblast	Dimitrovgrad	26-Sep-2020	Sivyakov V.	OQ078939
180.	NK-20-45	GPRU094-21	Russia	Ulyanovsk Oblast	Dimitrovgrad	26-Sep-2020	Sivyakov V.	OQ079024
181.	NK-20-46	GPRU095-21	Russia	Ulyanovsk Oblast	Dimitrovgrad	26-Sep-2020	Sivyakov V.	OQ078931
182.	VA-20-9	GPRU060-21	Russia	Ulyanovsk Oblast	Dimitrovgrad	20-Oct-2020	Sivyakov V.	OQ079054
183.	VA-20-31	GPRU082-21	Russia	Ulyanovsk Oblast	Dimitrovgrad	20-Oct-2020	Zolotuhin V.	OQ079048
184.	VA-20-32	GPRU083-21	Russia	Ulyanovsk Oblast	Dimitrovgrad	20-Oct-2020	Zolotuhin V.	OQ078941
185.	VZ-20-05	GPRU039-21	Russia	Ulyanovsk Oblast	Dimitrovgrad	26-Sep-2020	Sivyakov V.	OQ078923
186.	VZ-20-06	GPRU040-21	Russia	Ulyanovsk Oblast	Dimitrovgrad	26-Sep-2020	Sivyakov V.	OQ079061
187.	VZ-20-01	GPRU035-21	Russia	Ulyanovsk Oblast	Inza	02-Sep-2020	Ladonina D.	OQ079052
188.	VZ-20-03	GPRU037-21	Russia	Ulyanovsk Oblast	Inza	26-Sep-2020	Ladonina D.	OQ079059
189.	VZ-20-04	GPRU038-21	Russia	Ulyanovsk Oblast	Inza	29-Sep-2020	Ladonina D.	OQ079013
190.	VZ-20-07	GPRU041-21	Russia	Ulyanovsk Oblast	Karsun	26-Sep-2020	Zolotuhin V.	OQ079004
191.	VA-20-10	GPRU061-21	Russia	Ulyanovsk Oblast	Ulyanovsk	20-Sep-2020	Zolotuhin V.	OQ079008
192.	VA-20-11	GPRU062-21	Russia	Ulyanovsk Oblast	Ulyanovsk	20-Sep-2020	Zolotuhin V.	OQ078975
193.	VA-20-12	GPRU063-21	Russia	Ulyanovsk Oblast	Ulyanovsk	20-Sep-2020	Zolotuhin V.	OQ079092
194.	VA-20-26	GPRU077-21	Russia	Ulyanovsk Oblast	Ulyanovsk	20-Sep-2020	Zolotuhin V.	OQ078996
195.	VA-20-27	GPRU078-21	Russia	Ulyanovsk Oblast	Ulyanovsk	20-Sep-2020	Zolotuhin V.	OQ079090
196.	VA-20-28	GPRU079-21	Russia	Ulyanovsk Oblast	Ulyanovsk	20-Sep-2020	Zolotuhin V.	OQ078973
197.	NK1014	CAMRU099-21	Russia	Yaroslavl Oblast	Yaroslavl	20-Jul-2021	Vlasov D.	OQ078945
198.	NK1015	CAMRU100-21	Russia	Yaroslavl Oblast	Yaroslavl	20-Jul-2021	Vlasov D.	OQ078960
199.	NK1016	CAMRU101-21	Russia	Yaroslavl Oblast	Yaroslavl	20-Jul-2021	Vlasov D.	OQ078974

No.	Sample ID	Process ID	Country	Region	Place	Collection data	Collector	GenBank Accession
200.	NK1082	CAMRU167-21	Russia	Yaroslavl Oblast	Yaroslavl	20-Jul-2021	Vlasov D.	OQ079018
201.	NK1083	CAMRU168-21	Russia	Yaroslavl Oblast	Yaroslavl	20-Jul-2021	Vlasov D.	OQ079099
**Sequences borrowed from BOLD for comparison**								
202.	BIOUG36740-C07	GMBMN825-17	Belarus	Minskaya Voblasts	Minsk	07-Jul-2016	Lipinskaya T.	MW214209
203.	BIOUG36740-H05	GMBMN883-17	Belarus	Minskaya Voblasts	Minsk	07-Jul-2016	Lipinskaya T.	MW214334
204.	RV15d	CAMER019-07	France		Mezieres les clery	20-Sep-2005	Garcia J.	GQ144274
205.	CCDB-02228-H06	MICOW283-10	Greece	Thessaly	Larisa	06-Jun-2008	Lopez-Vaamonde C., Augustin S.	HM379297
206.	RV68	CAMER040-07	Hungary		Borjad	03-Dec-2006	Wagner M.	GQ144278
207.	DLCO112	LNOUD191-11	Macedonia			01-Aug-1985	Deschka G.	KX044715
**Outgroup**								
208.	NK529	SIBLE018-17	Russia	Primorskiy Krai	Gornotaejnoe village	25-Jul-2016	Kirichenko N.	MK403719
209.	NK537	SIBLE026-17	Russia	Primorskiy Krai	Gornotaejnoe village	22-Jul-2016	Kirichenko N.	MK403685
